# The efficacy and safety of short-course radiotherapy followed by sequential chemotherapy and Cadonilimab for locally advanced rectal cancer: a protocol of a phase II study

**DOI:** 10.1186/s12885-024-12254-1

**Published:** 2024-04-19

**Authors:** Tongzhen Xu, Lingling Feng, Wenjue Zhang, Haoyue Li, Huiying Ma, Muyasha Abulimiti, Yutong Tan, Feiyan Deng, Wenting Huang, Shuangmei Zou, Wenyan Kang, Liming Jiang, Ying Wang, Chen Hu, Yinggang Chen, Haitao Zhou, Yuan Tang, Jing Jin

**Affiliations:** 1 State Key Laboratory of Molecular Oncology and Department of Radiation Oncology, National Cancer Center/National Clinical Research Center for Cancer/Cancer Hospital, Chinese Academy of Medical Sciences and Peking Union Medical College, Beijing, 100021 China; 2 Department of Radiation Oncology, National Cancer Center/National Clinical Research Center for Cancer/Cancer Hospital & Shenzhen Hospital, Chinese Academy of Medical Sciences and Peking Union Medical College, Shenzhen, 518116 China; 3 Department of Pathology, National Cancer Center/National Clinical Research Center for Cancer/Cancer Hospital & Shenzhen Hospital, Chinese Academy of Medical Sciences and Peking Union Medical College, Shenzhen, 518116 China; 4 State Key Laboratory of Molecular Oncology and Department of Pathology, National Cancer Center/National Clinical Research Center for Cancer/Cancer Hospital, Chinese Academy of Medical Sciences and Peking Union Medical College, Beijing, 100021 China; 5 Department of Radiology, National Cancer Center/National Clinical Research Center for Cancer/Cancer Hospital & Shenzhen Hospital, Chinese Academy of Medical Sciences and Peking Union Medical College, Shenzhen, 518116 China; 6 State Key Laboratory of Molecular Oncology and Department of Radiology, National Cancer Center/National Clinical Research Center for Cancer/Cancer Hospital, Chinese Academy of Medical Sciences and Peking Union Medical College, Beijing, 100021 China; 7 Department of Medical Oncology, National Cancer Center/National Clinical Research Center for Cancer/Cancer Hospital & Shenzhen Hospital, Chinese Academy of Medical Sciences and Peking Union Medical College, Shenzhen, 518116 China; 8 Division of Biostatistics and Bioinformatics, Sidney Kimmel Comprehensive Cancer Center, Johns Hopkins University School of Medicine, Baltimore, USA; 9 Department of Colorectal Surgery, National Cancer Center/National Clinical Research Center for Cancer/Cancer Hospital & Shenzhen Hospital, Chinese Academy of Medical Sciences and Peking Union Medical College, Shenzhen, 518116 China; 10 State Key Laboratory of Molecular Oncology and Department of Colorectal Surgery, National Cancer Center/National Clinical Research Center for Cancer/Cancer Hospital, Chinese Academy of Medical Sciences and Peking Union Medical College, Beijing, 100021 China

**Keywords:** Locally advanced rectal cancer, Bispecific antibody immunotherapy, Short-course radiotherapy, Total neoadjuvant therapy, Complete response

## Abstract

**Background:**

For patients with locally advanced rectal cancer (LARC), total neoadjuvant therapy (TNT), namely, intensifying preoperative treatment through the integration of radiotherapy and systemic chemotherapy before surgery, was commonly recommended as the standard treatment. However, the risk of distant metastasis at 3 years remained higher than 20%, and the complete response (CR) rate was less than 30%. Several clinical trials had suggested a higher complete response rate when combining single-agent immunotherapy with short-course radiotherapy (SCRT). The CheckMate 142 study had shown encouraging outcomes of dual immunotherapy and seemingly comparable toxicity for CRC compared with single-agent immunotherapy in historical results. Therefore, dual immunotherapy might be more feasible in conjunction with the TNT paradigm of SCRT. We performed a phase II study to investigate whether the addition of a dual immune checkpoint inhibitor bispecific antibody, Cadonilimab, to SCRT combined with chemotherapy might further increase the clinical benefit and prognosis for LARC patients.

**Methods:**

This single-arm, multicenter, prospective, phase II study included patients with pathologically confirmed cT3-T4N0 or cT2-4N + rectal adenocarcinoma with an ECOG performance score of 0 or 1. Bispecific antibody immunotherapy was added to SCRT combined with chemotherapy. Patients enrolled would be treated with SCRT (25 Gy in five fractions over 1 week) for the pelvic cavity, followed by 4 cycles of CAPOX or 6 cycles of mFOLFOX and Cadonilimab. The primary endpoint was the CR rate, which was the ratio of the pathological CR rate plus the clinical CR rate. The secondary endpoints included local–regional control, distant metastasis, disease-free survival, overall survival, toxicity profile, quality of life and functional outcome of the rectum. To detect an increase in the complete remission rate from 21.8% to 40% with 80% power, 50 patients were needed.

**Discussion:**

This study would provide evidence on the efficacy and safety of SCRT plus bispecific antibody immunotherapy combined with chemotherapy as neoadjuvant therapy for patients with LARC, which might be used as a candidate potential therapy in the future.

**Trial registration:**

This phase II trial was prospectively registered at ClinicalTrials.gov, under the identifier NCT05794750.

**Supplementary Information:**

The online version contains supplementary material available at 10.1186/s12885-024-12254-1.

## Introduction

For patients with locally advanced rectal cancer (LARC), preoperative long-course radiotherapy or chemoradiotherapy (CRT) combined with total mesorectal excision (TME) had been considered as the standard treatment, which greatly decreased local recurrence and improves the tumor resection rate [1, 2]. Short-course radiotherapy (SCRT) with preoperative chemotherapy followed by surgery was also deemed as an efficacious alternative to CRT for LARC. Even though the local recurrence rate had plummeted to less than 10% in the era of gradually increasing adoption of neoadjuvant chemoradiotherapy plus TME surgery for LARC, a considerable proportion of patients with nonmetastatic rectal cancer, especially those with high-risk features, were still at high risk of suffering from distant metastasis with an incidence ranging from 26 to 36% [3–5].

Total neoadjuvant therapy (TNT), standing out as a promising approach, aimed to improve overall survival (OS) by intensifying preoperative treatment to eliminate early micrometastatic lesions through the integration of radiotherapy and systemic chemotherapy before surgery. Randomized studies on TNT for LARC had demonstrated improved rates of distant metastasis-free survival (DMFS) and increased tumor complete response (CR) rates [6]. A better pathological CR (pCR) rate (28% vs 14%, *P* < 0.0001) and improved DMFS [7] were observed in another phase III trial (RAPIDO) for LARC patients treated with SCRT and preoperative chemotherapy. Furthermore, the STELLAR study showed that a TNT paradigm of SCRT followed by neoadjuvant chemotherapy could achieve CR rates (21.8% vs 12.3%, *P* = 0.002) superior to long-course CRT, without compromising on 3-year disease-free survival (DFS) [8]. At present, most studies had shown that the treatment strategy of TNT rather than conventional CRT could achieve better tumor control and a higher CR rate for LARC, especially for those with high-risk features, resulting in increasing possibilities of rectal organ preservation and satisfying quality of life. However, the risk of distant metastasis at 3 years remained higher than 20%, and the CR rate was less than 30% [7, 8].

How to maintain a high CR rate while reducing the distant metastasis rate was challenging and had fallen under spotlight for years. Accumulating evidences had suggested that radiotherapy might also eliminate tumors by activating local and/or systemic immune responses, especially when it was combined with immunotherapy such as immune checkpoint inhibitors [9]. There was a synergistic sensitizing effect when radiotherapy was combined with immune checkpoint inhibitors. Radiotherapy would promote the release of tumor-specific antigens and upregulate the expression of major histocompatibility complex (MHC) on cancer cells to promote tumor antigen presentation to cytotoxic T cells, and it would be easier for immunotherapy drugs to recognize these antigens and facilitate antigen-presenting cells to phagocytize damaged tumor cells [10]. In addition, radiotherapy could result in the reshaping of the tumor microenvironment, reduce immunosuppressive factors and increase the infiltration of effector T cells [11]. Immunotherapy might improve radiosensitivity by normalizing tumor vascularity and hypoxia [12]. There was still a large space worthy of further exploration for the treatment of combining radiotherapy and immunotherapy.

For radiotherapy, most LARC clinical trials were designed to explore the efficacy of combining preoperative long-course CRT with single agent immunotherapy in the TNT setting, some of which had released their final results of pCR rates of 24–32.7% [13–15]. Instead of long-course CRT, SCRT combined with immunotherapy might further improve the pCR rate, because substantial immune-stimulatory potential had been observed in rectal cancer treated with SCRT [16]. The Averectal trial showed that SCRT followed by mFOLOFX6 and Avelumab had an acceptable toxicity profile and a 37.5% pCR rate for LARC, which was better than standard neoadjuvant treatment [17]. Another phase II trial (NCT04231552) also reported a pCR rate of 100% in rectal cancer patients with deficient mismatch repair/microsatellite instability-high (dMMR/MSI-H) and 46% in patients without mismatch repair defects [18].

For immunotherapy, several clinical studies had demonstrated that combination therapy of anti-cytotoxic T-lymphocyte antigen-4 (CTLA-4) antibodies and anti-programmed cell death protein-1 (PD-1) antibodies could significantly improve oncological outcomes for some hard-to-treat cancer types. In the first-line treatment of metastatic colorectal cancer (CRC) patients, the dual immunotherapy combination regimen from the CheckMate 142 study and MEDITREME trial had shown high objective response rates (ORR) of 69% and 64.5%, with encouraging progression-free survival and OS at 24 months (74% and 79%, 6.7% and 57.6%, respectively) [19, 20]. In the background of preoperative treatment, the NICHE trial had also indicated that neoadjuvant dual immunotherapy could improve pathological response outcome for early-stage CRC with a pCR rate of 60% in dMMR tumors [21]. However, severe toxicities hampered the further application of dual immunotherapy [19–22].

Cadonilimab, as an immunotherapy agent, was the first anti-PD-1/CTLA-4 bispecific antibody approved in China for relapsed or metastatic cervical cancer [23]. A phase Ia/Ib study on relapsed mesothelioma showed tolerability and encouraging antitumor activity of Cadonilimab [24]. Several phase Ib/II studies on advanced solid tumors, including cervical cancer, esophageal squamous cell carcinoma and hepatocellular carcinoma, had also demonstrated the efficacy and safety of this novel bispecific antibody [25–30]. Anti-PD-1/CTLA-4 bispecific antibody might be more feasible in conjunction with SCRT for LARC. We hypothesized that the addition of a dual immune checkpoint inhibitor bispecific antibody, Cadonilimab, to SCRT combined with chemotherapy might further increase the clinical benefit and prognosis for LARC patients.

## Methods/design

### Trial design

**Fig. 1:**
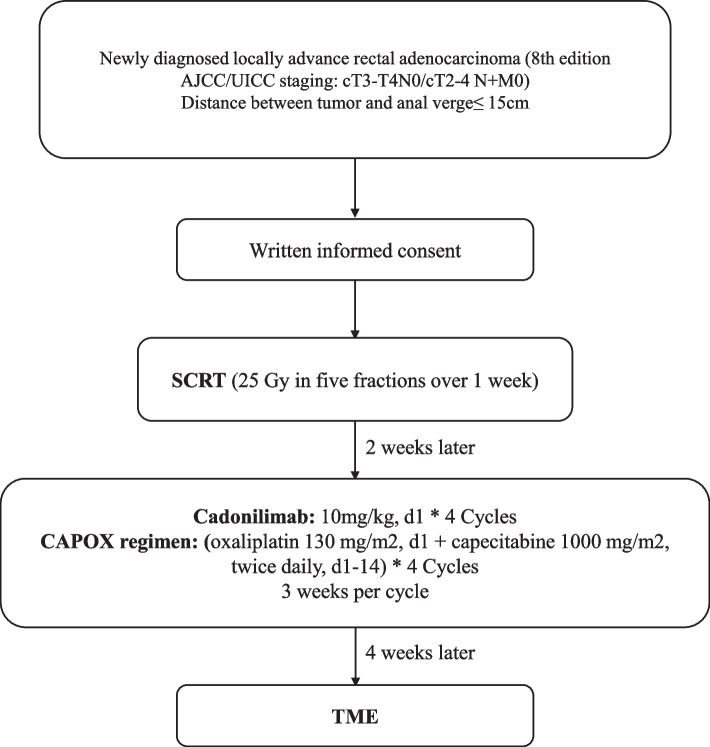
Study design. Abbreviation: AJCC. American Joint Committee on Cancer; UICC. Union for International Cancer Control; SCRT. Short-course radiotherapy; TME. Total mesorectal excision; Note: The mFOLFOX regimen is an alternative for those who are intolerable with CAPOX regimen. mFOLFOX regimen: (oxaliplatin 85 mg/m2 d1, leucovorin 400 mg/m2 d1 + fluorouracil 400 mg/m2 d1 followed by fluorouracil 2400 mg/m2 as a 44 to 46 hours infusion) * 6 Cycles, 2 weeks per cycle

This was a phase II, multicenter, single-arm, open-label, prospective study (ClinicalTrials.gov Identifier: NCT05794750) for cT3-T4N0 or cT2-4N + rectal cancer. Bispecific antibody immunotherapy was added to SCRT combined with chemotherapy. The study was conducted in accordance with the Declaration of Helsinki and Good Clinical Practice. The current protocol was version2.0 dated October 8, 2022. The protocol and its amendments had been approved by the ethics committee of the Cancer Hospital&Shenzhen Hospital Chinese Academy of Medical Sciences (YW2022-21–3) and the Cancer Hospital Chinese Academy of Medical Sciences (23/283–4025). The bispecific antibody, Cadonilimab, was supported by Akesobio. Response Evaluation Criteria in Solid Tumors (RECIST) Version1.1, National Cancer Institute Common Terminology Criteria for Adverse Events (NCI-CTCAE) Version5.0 and Eastern Cooperative Oncology Group (ECOG) were adopted to evaluate the tumor response, adverse events (AEs) and performance status, respectively [31–33]. The study design was summarized in Fig. 1.

### Eligibility criteria

The inclusion and exclusion criteria for patients were listed in Supplementary Table 1.

## Objectives

### Primary endpoint

- Percent of patients achieving CR, which was the ratio of pCR rate plus clinical complete response (cCR) rate. A pCR was defined as the absence of tumor cells at the primary site and regional lymph nodes. For the precise judgement and diagnosis of cCR, the cCR criteria set by Mass in 2011 were applied [34] and were shown in Table 1.

**Table 1:** Criteria for clinical complete response

Diagnosis criteria
1. Substantial downsizing with no residual tumor or residual fibrosis only (with low signal on high b-value DWI, if available) 2. No suspicious lymph nodes on MRI 3. No residual tumor at endoscopy or only a small residual erythematous ulcer or scar 4. Negative biopsies from the scar, ulcer, or former tumor location 5. No palpable tumor, when initially palpable with digital rectal examination

*Abbreviation*: *DWI* Diffusion weighted imaging, *MRI* Magnetic resonance imaging

If patients did not meet all of these criteria, the diagnosis of clinical complete response could not be validated

### Secondary endpoints

- 3-year local–regional recurrence-free survival (LRFS) rate (LRFS was defined as the time from the date of radiation to the first recurrence or progression of tumor within its original site or surrounding region)
- 3-year DMFS rate (DMFS was defined as the time from the date of radiation to the first occurrence of distant metastasis)
- 3-year DFS rate (DFS was defined as the time from the date of radiation to the first occurrence of local–regional failure, distant metastasis, second primary tumor, or death from any cause)
- 3-year OS rate (OS was defined as the time from the date of radiation to death from any cause)
- Toxicity profile (NCI-CTCAE v5.0)
- Assessment of quality of life (QoL) and functional outcome of the rectum

### Exploratory endpoints

- The correlation between the expression of immune markers and the distribution of immune cells in tumor tissue or blood and the clinical outcome. These included the application of single-cell transcriptomics and spatial transcriptomics to identify the characteristics of immune cells within the tumor microenvironment, the utilization of Proximity Extension Assay by Olink Proteomics to explore multiple cytokine changes at the protein level and the investigation of the dynamic alteration of peripheral blood mononuclear cells using Cytometry by Time-of-Flight technology.
- The relationship between circulating tumor DNA and the outcome of patients.

## Intervention

### Neoadjuvant treatment modality

Patients enrolled would be treated with SCRT (25 Gy in five fractions over 1 week) for the pelvic cavity, followed by 4 cycles of CAPOX or 6 cycles of mFOLFOX and Cadonilimab. The CAPOX regimen included oxaliplatin 130 mg/m2 d1, and capecitabine 1000 mg/m2 bid, d1-14 (3 weeks per cycle). The mFOLFOX regimen was an alternative for those who could not tolerate with CAPOX regimen. It included oxaliplatin 85 mg/m2 d1, leucovorin 400 mg/m2 d1 plus fluorouracil 400 mg/m2 d1 followed by fluorouracil 2400 mg/m2 as a 44 to 46 h infusion, repeated every 2 weeks. Cadonilimab (10 mg/kg d1) was administered for 4 cycles (3 weeks per cycle). All patients received intensity-modulated radiation therapy (IMRT), volume modulated arc therapy (VMAT) or tomotherapy (TOMO). Before rectal cancer radiotherapy, patients underwent preparatory measures for accurate positioning. These included emptying the rectum as much as possible, with the option of using stool softeners like liquid paraffin if necessary. One hour before positioning, patients voided their bladder and drink 1000ml of water without voiding again to ensure bladder filling. Lead markers were also positioned at the anal verge to aid localization during treatment. CT-based planning was conducted with axial images taken at intervals of 5 mm, covering the upper lumbar spine to the mid-femur and MR scan was also collected on the same positioning condition for fusion with the planning CT-scan. Intravenous contrast was required to enhance the visualization of the iliac and inguinal vessels. The clinical target volume (CTV) included the primary tumor, regional lymph nodes, and pelvic regions at risk according to our previous STELLAR study. The mesorectum, presacral space, internal iliac nodes, obturator nodes, and ischiorectal fossa were covered within the CTV, and if the rectal tumor was staged as T4b, external iliac nodes should be included. The superior border was defined as the sacral promontory. The inferior border was 2–3 cm distal to the lower pole of the tumor. Expansion of the CTV to the planning target volume (PTV) was 0.5–1.0 cm.

During treatment, Cadonilimab dose adjustment was not allowed, but delayed doses were permitted for up to 12 weeks. Immune-related adverse events (irAEs) were defined as adverse events related to immune checkpoint inhibitors, consistent with immune-mediated mechanisms and unable to be attributed to other certain causes. Criteria for Cadonilimab pausing and discontinuing when specific irAEs appeared in the neoadjuvant treatment were shown in Supplementary Table 2. For irAEs unmentioned above or some special toxicities, the practice guidelines of the management of immune-related adverse events in patients treated with immune checkpoint inhibitors would be referenced [35]. For investigator-assessed grade 3 or higher non-irAEs associated with Cadonilimab, the administration of Cadonilimab could be withheld for clinical management, and the decision to continue medication could be made at the discretion of the investigator until the adverse events had resolved or improved. Dose interruptions were unnecessary for AEs that were clearly unrelated to cardonilimab or for laboratory abnormalities that were not clinically significant. All toxicities were graded according to NCI-CTCAE v5.0.

Criteria for dose adjustment of neoadjuvant chemotherapy agents when chemotherapy-related AEs appeared were shown in Supplementary Table 3. Dose adjustments in chemotherapy should be based on the maximum graded toxicity within the previous cycle. For patients who suffered from hematological toxicity and/or non-hematological toxicity for more than 21 days, chemotherapy should be discontinued unless the investigator and sponsor reached an agreement on continuation due to potential clinical benefit for patients. If a decrease in white blood cells or neutrophils of grade 3 or 4 emerged, chemotherapy should be withheld and the original dose could be maintained if it recovered to grade 1 or baseline level at 21 days. If febrile neutropenia occurred (neutrophils < 1000 per cubic millimeter, with fever of ≥ 38° C for more than 1 h or a single temperature measurement of > 38.3° C) or grade 4 neutropenia occurred repeatedly or for up to 7 days, dose reductions were required after recovering to grade 1 or baseline levels at 21 days. In addition, nausea, vomiting, or diarrhea was initially treated with supportive care, and if this was still unacceptable, chemotherapy was withheld until toxicity recovered to grade 1 or baseline levels. For the side effects that were considered by the investigators to be unlikely to develop into serious or life-threatening AEs (hyperuricemia, hypophosphatemia, alopecia, etc.) and could be tolerated by the patients, the chemotherapy could be maintained at the same dose level without dose reduction or interruption, as long as the patients were actively given symptomatic treatment.

Radiotherapy should be paused when there was grade 3 or higher radiation dermatitis in the radiation field and restarted when dermatitis recovered to grade 1 or greater. Radiotherapy should also be paused if any grade 4 AEs occurred and radiotherapy could be recontinued when the AEs recovered to grade 1 or greater. The prescribed dose of radiotherapy was not amended unless new AEs occurred or the original AEs were aggravated.

### Radical surgery

Four weeks after the final cycle of preoperative chemotherapy combined with bispecific antibody immunotherapy, a multidisciplinary team (MDT) meeting, including surgeon, radiation oncologist, medical oncologist, radiologist, pathologist and so forth, would be launched and image evaluation will be adopted for patients to confirm whether R0 resection was possible and if cCR criteria were reached. If R0 resection was feasible, surgery would be performed 28 days after the first day of the last administration of chemotherapy combined with bispecific antibody immunotherapy in the final course. If R0 resection was infeasible or disease progression was observed, the protocol treatment will be discontinued. The surgery must be a total mesorectal excision, and the exact procedure would be decided by the surgeon, including anterior resection (AR), laparoscopic resection (APR) or Hartmann's procedure. Extending 4 to 5 cm below the distal edge of tumors for an adequate mesorectal excision was needed. A negative margin of 1 to 2 cm for distal rectal cancers (< 5 cm from anal verge) was acceptable. For patients with suspicious lateral pelvic lymph node involvement, whether to perform extended resections beyond the TME plane depended on the actual situation and MDT advice. The expression of MMR protein and PD-1 immunohistochemical results should be described in the preoperative biopsy report. The postoperative pathological diagnosis should include the gross condition of the tumor, histological type and grade, treatment response, resection margin, lymph node metastasis, pathological stage and immunohistochemical results. If a patient reached the cCR criteria, the cCR follow-up strategy was carried out.

### Follow-up after treatment completion

Patients would be followed up on a fixed schedule for at least three years after surgical completion. The first and second visits would be arranged 30 days and 90 days after resection to evaluate the safety and tolerability of treatment, and then every 3 months thereafter to document the survival outcome of each patient. See Supplementary Table 4 and Supplementary Table 5 for the schedule of evaluations after the completion of treatment. Each patient remained on study until disease progression, infeasibility of surgery after completion of neoadjuvant treatment, withdrawal of consent, unacceptable toxicity, pregnancy, loss of follow-up, or death. Patients with cCR after preoperative chemoradiotherapy and immunotherapy and then firmly choosing the wait and watch strategy would be followed up according to an intensively follow-up strategy in every 3 months requirement of comprehensive examination, including pelvic magnetic resonance imaging (MRI), computed tomography (CT) imaging, colorectal endoscopy, digital rectal examination and routine laboratory tests.

### Data collection of QoL and rectal function

During the protocol treatment and follow-up period, the European Organization for Research and Treatment of Cancer (EORTC) questionnaires QLQ-C30 [36, 37] and CR29 [38] were adopted as patient reported outcome measures (PROMs) to assess QoL at baseline, before and 1 month after surgery, and 6, 12, 24 and 36 months after surgery. The functional outcome of the rectum would be collected through the Wexner score [39] for patients who received sphincter-preserving surgery at the same time as the assessment of QoL. Specifically, for patients with cCR, QoL and functional outcome of the rectum 1 month after the date of confirming cCR would be collected, and an intensive every 3 months schedule of the assessment of QoL and functional outcome of the rectum would be carried out after the date of confirming cCR in the first year and then every 6 months in the second and third years.

### Safety run-in analysis

Due to a lack of data about the safety profile of combining Cadonilimab with SCRT and chemotherapy for LARC, a safety run-in phase was conducted to evaluate the feasibility of the experimental treatment. A total of 6 patients were included according to the eligibility criteria. The safety profile was reviewed by an Independent Data Monitoring Committee (IDMC). It was gathered 28 days after the 6th patient received the first cycle of Cadonilimab plus chemotherapy. If the first 6 patients tolerated this combination treatment and no safety issues arose, the enrollment would be continued.

### Statistical analysis for primary endpoint

Based on the results of a previous study [8], the objective of this study was to increase the complete remission rate from 21.8% to 40%. A Simon two-stage approach optimal design was used to yield a type-1 error of 0.05 and 80% power. The first phase would include 8 patients, plus 6 patients in the safety run-in period, for a total of 14 patients, and if ≥ 4 of these patients achieved a CR, the study would move into the second phase. Taking into account for a 10% drop-out rate, the total actual goal was 50 patients.

Following the intent-to-treat principle, the full analysis set, defined as all patients who received at least one dose of the study drug with measurable lesions at baseline according to RECIST v1.1, would be utilized to conduct the efficacy analysis. At the same time, the safety set, defined as all patients who received at least one dose of study drug according to the actual adoption of treatment for patients, would be utilized to conduct the safety analysis. Descriptive statistics would be conducted on baseline characteristics, tumor response, biomarkers, and AEs. Survival curves would be plotted using the Kaplan–Meier method. The missing data would not be included in the final data analysis.

### Monitoring

Regular monitoring would be performed annually by an IDMC to verify the protocol compliance, the accuracy and consistency of the data and the adherence to the International Conference on Harmonization-Good Clinical Practice (ICH-GCP) and local regulations when conducting the clinical study.

### Status of the trial

This trial started in April 2023 and patient recruitment was ongoing on August 30, 2023.

## Discussion

This study would provide evidence on the efficacy and safety of SCRT plus bispecific antibody immunotherapy combined with chemotherapy as neoadjuvant therapy for patients with LARC, which might be used as a candidate potential therapy in the future.

A preclinical study showed that the immune response induced by radiation might be dose-dependent [40]. The immune response induced by high-dose irradiation was different from that induced by conventionally fractionated irradiation. High dose irradiation could induce the activation of the death receptor Fas signaling pathway, which rendered irradiated cells susceptible to killing by cytotoxic lymphocytes [41]. Reits et al. [42] found that ≥ 10 Gy radiation could increase the expression of MHC-1 and induce an antitumor immune response in mice with colon cancer. A recent randomized phase II trial showed that, compared to stereotactic ablative radiotherapy (SABR) alone, SABR combined with immunotherapy could significantly improve event-free survival from 53 to 77% in early-stage non-small cell lung cancer [43]. As the radiation dose increased, this phenomenon might be more obvious while low-dose radiation had no such effect. The upregulation in MHC-1 expression induced by high-dose radiation was due to the induction and activation of mammalian target of rapamycin (mTOR), since rapamycin (an mTOR inhibitor) could reduce the radiation-induced high expression of MHC-1. The application of SCRT in this study might be able to further promote the immune response and tumor regression.

The utilization of dual immunotherapy was a potential research direction for improving the prognosis of various types of solid tumors. The CheckMate 067 trial had shown a superior clinical benefit in advanced melanoma patients treated by nivolumab plus ipilimumab with a median OS of 72.1 months compared to nivolumab alone or ipilimumab alone [44]. Furthermore, a durable clinical outcome after the administration of dual immunotherapy was observed in patients with recurrent small cell lung cancer or metastatic urothelial carcinoma in the CheckMate 032 trial [45, 46]. Specifically, the results from a phase Ia/Ib study of an anti-CTLA-4 monoclonal antibody, plus an anti-PD-1 antibody in metastatic microsatellite stable (MSS) CRC yielded an objective response rate of 22% [47], while previous clinical studies had shown that MSS CRC did not benefit much from immunotherapy as a single agent [48]. For mCRC patients with MSI-H/dMMR, the anti-CTLA-4 plus anti-PD-1 regimen from the CheckMate 142 study was also better than the single anti-PD-1 regimen from the KEYNOTE 177 study [49, 50]. Regardless of microsatellite status, dual immunotherapy regimens might be more effective than monotherapy.

It was one of our main concerns that whether the addition of bispecific antibody into SCRT in this study increases adverse events. A phase Ib/II, multicenter study recently updated their two-year data of an ORR of 68.2%, with 5 (5.7%) complete responses and 55 (62.5%) partial responses in advanced gastric or gastroesophageal junction cancer patients treated with Cadonilimab and chemotherapy [51]. Grade ≥ 3 treatment-related adverse events occurred in 69.4% of patients, most of which were myelosuppression and gastrointestinal reactions without new safety signals identified. This preferable treatment outcome was replicated in advanced cervical cancer with an ORR of 68–92% and grade ≥ 3 treatment-related toxicity, mainly consisting of hematological toxicity, occurred in 51.1% of patients, whose grade ≥ 3 irAEs were reported with an incidence of 17.8% [52]. Based on the results, the treatment-related toxicity of adding Cadonilimab, into TNT was considered manageable and acceptable.

In summary, the combination with radiotherapy had the potential to improve the efficacy of immune checkpoint inhibitors. Therefore, the addition of bispecific antibodies to SCRT in the treatment strategy of TNT was worthy of further exploration of the rate of complete response and prognosis for LARC patients. We looked forward to obtaining better results and selecting a better treatment strategy.

### Supplementary Information

**Supplementary Material 1. **

**Supplementary Material 2. **

**Supplementary Material 3. **

**Supplementary Material 4. **

**Supplementary Material 5. **

## Data Availability

The datasets used and/or analyzed during the current study were available from the corresponding author upon reasonable request.

## Abbreviations

LARC: Locally Advanced Rectal Cancer
CRC: Colorectal Cancer
CRT: Chemoradiotherapy
TME: Total Mesorectal Excision
SCRT: Short-Course Radiotherapy
TNT: Total Neoadjuvant Therapy
CR: Complete Response
pCR: Pathological Complete Response
cCR: Clinical Complete response
LRFS: Local–Regional Recurrence-Free Survival
DMFS: Distant Metastasis-Free Survival
DFS: Disease-Free Survival
OS: Overall Survival
MHC: Major Histocompatibility Complex
dMMR: Deficient Mismatch Repair
MSI-H: Microsatellite Instability-High
CTLA-4: Cytotoxic T-Lymphocyte Antigen-4
PD-1: Programmed Cell Death Protein-1
ORR: Objective Response Rate
RECIST: Response Evaluation Criteria in Solid Tumors
NCI-CTCAE: National Cancer Institute Common Terminology Criteria for Adverse Events
AEs: Adverse Events
ECOG: Eastern Cooperative Oncology Group
*EORTC*: European Organization for Research and Treatment of Cancer
QoL: Quality of Life
CAPOX: Capecitabine, Oxaliplatin
FOLFOX: 5-Fluorouracil, Oxaliplatin
IMRT: Intensity Modulated Radiation Therapy
VMAT: Volume Modulated Arc Therapy
TOMO: Tomotherapy
CTV: Clinical Target Volume
PTV: Planning Target Volume
irAEs: Immune-Related Adverse Events
MDT: Multidisciplinary Team
AR: Anterior Resection
APR: Laparoscopic Resection
MRI: Magnetic Resonance Imaging
DWI: Diffusion Weighted Imaging
CT: Computed Tomography
IDMC: Independent Data Monitoring Committee
ICH-GCP: International Conference on Harmonization-Good Clinical Practice
SABR: Stereotactic Ablative Radiotherapy
mTOR: Mammalian Target of Rapamycin
MSS: Microsatellite Stable
ALT: Alanine Aminotransferase
AST: Aspartate Aminotransferase
ALP: Alkaline Phosphatase
TBIL: Total Bilirubin
HIV: Human Immunodeficiency Virus
HBV: Hepatitis B Virus
HCV: Hepatitis C Virus
