# Supplementary material for: The efficacy and safety of short-course radiotherapy followed by sequential chemotherapy and Cadonilimab for locally advanced rectal cancer: a protocol of a phase II study
Source: BMC Cancer. 2024 Apr 19;24:501. doi: 10.1186/s12885-024-12254-1 (PMC11031930; doi:10.1186/s12885-024-12254-1)
Supplement: Supplementary file 1 — Supplementary Material 1. [file 12885_2024_12254_MOESM1_ESM.docx]

**Supplementary Table 1: Inclusion and exclusion criteria for patients**

| Inclusion criteria | Exclusion criteria |
| --- | --- |
| 1. Biopsy proven rectal adenocarcinoma; 2. Distance between tumour and anal verge≤ 10 cm; 3. Locally advanced tumour;(8th edition AJCC/UICC staging: cT3-T4N0/cT2-4 N+M0) Cancer Staging must be based on pelvic MRI and Endoscopic ultrasound; 4. Eastern Cooperative Oncology Group (ECOG) performance score ≤ 1; 5. Mentally and physically fit for chemotherapy; Adequate blood counts:    1. White blood cell count ≥3.5 x 10^9^/L Haemoglobin levels ≥100 g/L    2. Platelet count ≥100 x 10^9^/L    3. Creatinine levels ≤1.0×UNL    4. Urea nitrogen levels ≤1.0×UNL    5. Alanine aminotransferase (ALT) ≤1.5×UNL    6. Aspartate aminotransferase (AST) ≤1.5×UNL    7. Alkaline phosphatase (ALP) ≤1.5×UNL    8. Total bilirubin (TBIL)≤1.5×UNL 6. No excision of tumour, chemotherapy or other antitumour treatment after the diagnosis. 7. No previous pelvic radiation history 8. Written informed consent | 1. Previous treatment with anti-PD-1/L1 and anti-CTLA-4 or other immune experimental drugs. 2. Severe autoimmune disease: active inflammatory bowel disease (including Crohn's disease, ulcerative colitis), rheumatoid arthritis, scleroderma, systemic lupus erythematosus, autoimmune vasculitis (e.g. Wegener's granulomatosis) 3. Symptomatic interstitial lung disease or active infectious/non-infectious pneumonia. 4. At risk for bowel perforation: active diverticulitis, intra-abdominal abscess, gastrointestinal obstruction, abdominal cancer or other known risk factors for bowel perforation. 5. History of other malignancies, excluding curable non-melanotic skin cancer and cervix carcinoma in situ 6. Active infection, heart failure, heart attack within 6 months, unstable angina or unstable arrhythmia. 7. Any condition investigator considered to possibly interfere with the results or place the patient at increased risk of treatment complications, or other uncontrollable disease. 8. Pregnancy or breast feeding 9. Immunodeficiency disorders including human immunodeficiency virus (HIV), or history of organ transplantation, allogeneic stem cell transplantation 10. Active hepatitis B virus (HBV) hepatitis (HBV-DNA ≥ 2000 U/mL), hepatitis C virus (HCV) hepatitis, active tuberculosis infection. 11. Oncology vaccination history or any vaccination within 4 weeks prior to the start of treatment. (Note: influenza vaccines are mostly inactivated and therefore allowed, intranasal preparations are usually live attenuated vaccines and therefore not allowed) 12. Concomitant other immune agents, chemotherapeutic agents, other drugs in clinical studies, and long-term cortisol application |
