# Supplementary material for: The efficacy and safety of short-course radiotherapy followed by sequential chemotherapy and Cadonilimab for locally advanced rectal cancer: a protocol of a phase II study
Source: BMC Cancer. 2024 Apr 19;24:501. doi: 10.1186/s12885-024-12254-1 (PMC11031930; doi:10.1186/s12885-024-12254-1)
Supplement: Supplementary file 2 — Supplementary Material 2. [file 12885_2024_12254_MOESM2_ESM.docx]

**Supplementary Table 2: Criteria for cadonilimab pausing and discontinuing when irAEs appear**

| **irAEs (NCI CTCAE v5.0)** | **Cadonilimab pausing** | **Cadonilimab** **discontinuing** |
| --- | --- | --- |
| Pneumonitis | Grade 2 | Grade 3-4, or recurring Grade 2 |
| Diarrhea/enteritis | Grade 2-3 | Grade 4 |
| AST/ALT or bilirubin increased | Grade 2 | Pausing or discontinuing for Grade 3 as per investigator’s discretion;  Grade 4 |
| Hypophysitis | Grade 2 | Pausing or discontinuing for Grade 3-4 as per investigator’s discretion |
| Hyperthyroidism | Grade 2 | Pausing or discontinuing for Grade 3-4 as per investigator’s discretion |
| Hypothyroidism | Continuing administration of cadonilimab for Grade 2-4 | |
| Nephritis and renal insufficiency | Grade 2 | Grade 3-4 |
| Myocarditis | Grade 1 | Pausing or discontinuing for Grade 2 as per investigator’s discretion;  Grade 3-4 |
| Other irAEs | Intolerable or persistent Grade 2 adverse events | Pausing or discontinuing for Grade 3 as per investigator’s discretion;  Grade 4 or recurring Grade 3 |
| Abbreviation: NCI CTCAE. National Cancer Institute Common Terminology Criteria for Adverse Events; irAEs. immune-related adverse events; ALT. alanine aminotransferase; AST. aspartate aminotransferase;  Note:  1. The decision to suspend or permanently discontinue the study regimen was made after discussion between the investigator and the medical monitor.  2. For cadonilimab discontinuation: if AE returns to grade 0-1 or baseline and corticosteroids dose tapering (≤10mg of prednisone or equivalent) was completed, and restart of cadonilimab was allowed. If the AE did not recover within 12 weeks after the last administration of immunotherapy or the corticosteroids dose could not be reduced to ≤10mg prednisone or equivalent within 12 weeks, the immunotherapy was discontinued permanently.  3. For severe and life-threatening irAEs, intravenous corticosteroids should be initiated, followed by oral corticosteroids. If corticosteroids are not effective, other immunosuppressive agents should be considered.  4. Patients who developed grade 3-4 immune-related endocrine disease requiring suspension of cadonilimab were allowed to resume administration when AE recovered to ≤ grade 2 and was controlled by hormone replacement therapy. | | |
