# Supplementary material for: The efficacy and safety of short-course radiotherapy followed by sequential chemotherapy and Cadonilimab for locally advanced rectal cancer: a protocol of a phase II study
Source: BMC Cancer. 2024 Apr 19;24:501. doi: 10.1186/s12885-024-12254-1 (PMC11031930; doi:10.1186/s12885-024-12254-1)
Supplement: Supplementary file 3 — Supplementary Material 3. [file 12885_2024_12254_MOESM3_ESM.docx]

**Supplementary Table** **3: Criteria for dose adjustment of neoadjuvant chemotherapy agents when chemotherapy-related AEs appear**

| **Chemotherapy-related AEs** | **Grade (NCI CTCAE v5.0)** | **Dose adjustment** |
| --- | --- | --- |
| Hematological toxicities | Grade 1 | Observation while administering chemotherapy as planned initially |
|  | Grade 2 | Observation or starting symptomatic treatment while administering chemotherapy as planned initially |
|  | Grade 3-4 (For those considered manageable or reversible after dose reduction) | Suspending chemotherapy and starting symptomatic treatment until recovering to Grade 1 or baseline level |
|  | Grade 3-4 (For those considered unmanageable or irreversible after dose reduction) | Suspending chemotherapy and starting symptomatic treatment until recovering to Grade 1 or baseline level, or discontinuing chemotherapy |
|  | Grade 3-4 (For those taking 21 days to recover to Grade 1 or baseline) | Reducing by 10% to 15% of the previous dose level |
|  | recurring Grade 3 | Reducing by 10% to 15% of the previous dose level or discontinuing chemotherapy |
|  | recurring Grade 4 | Discontinuing chemotherapy |
| Non-hematological toxicities | Grade 1-2 (tolerable) | Observation while administering chemotherapy as planned initially |
|  | Grade 2 (intolerable) | Suspending chemotherapy and no dose adjustment if it recovers to Grade 1 or baseline level within 21 days |
|  | Grade 3-4 (For those considered manageable or reversible after dose reduction) | Suspending chemotherapy and Reducing by 10% to 15% of the previous dose level if it recovers to Grade 1 or baseline level within 21 days |
|  | Grade 3-4 (For those considered unmanageable or irreversible after dose reduction) | Discontinuing chemotherapy |
|  | recurring Grade 2 | Reducing by 10% to 15% of the previous dose level |
|  | recurring Grade 3 | Reducing by 10% to 15% of the previous dose level or discontinuing chemotherapy |
|  | recurring Grade 4 | Discontinuing chemotherapy |
