# Supplementary material for: The efficacy and safety of short-course radiotherapy followed by sequential chemotherapy and Cadonilimab for locally advanced rectal cancer: a protocol of a phase II study
Source: BMC Cancer. 2024 Apr 19;24:501. doi: 10.1186/s12885-024-12254-1 (PMC11031930; doi:10.1186/s12885-024-12254-1)
Supplement: Supplementary file 4 — Supplementary Material 4. [file 12885_2024_12254_MOESM4_ESM.docx]

**Supplementary Table 4: The schedule of evaluations after treatment completion for patients who receive surgery**

|  | 30 days  after surgery | 90 days  after surgery | Every 3 months thereafter |
| --- | --- | --- | --- |
| **Clinical examination and evaluation** | | | |
| Toxicity assessments | X | X |  |
| The usage of drug support therapy  and expectant treatment | X | X |  |
| Physical examination | X | X | X |
| Digital rectal examination | X | X | X |
| Vital signs | X | X |  |
| ECOG performance status | X | X |  |
| **Sample collection for biomarker** | | | |
| Peripheral blood sample | Blood samples will be collected at 3 months, 1 year (if feasible), and 2 years (if feasible) after surgery | | |
| **Imaging evaluation** | | | |
| Enhanced CT (required) or enhanced MRI (if needed) | X | X | X |
| **Laboratory examination** |  |  |  |
| Blood routine examination | X | X |  |
| Biochemical test | X | X |  |
| Routine urine test | X | X |  |
| Thyroid function | X | X |  |
| Cardiac marker  (cardiac enzymes, troponin) | X | X |  |
| Survival follow-up and subsequent antitumor therapy |  | X | X |
| **Quality-of-life and rectal function assessments** | X | X (assessing at 6, 12, 24 and 36 months after surgery) | |
