# Supplementary material for: The efficacy and safety of short-course radiotherapy followed by sequential chemotherapy and Cadonilimab for locally advanced rectal cancer: a protocol of a phase II study
Source: BMC Cancer. 2024 Apr 19;24:501. doi: 10.1186/s12885-024-12254-1 (PMC11031930; doi:10.1186/s12885-024-12254-1)
Supplement: Supplementary file 5 — Supplementary Material 5. [file 12885_2024_12254_MOESM5_ESM.docx]

**Supplementary Table 5: The schedule of evaluations after treatment completion for cCR patients**

|  | 30 days after choosing wait and watch strategy | 90 days after choosing wait and watch strategy | Every 3 months thereafter |
| --- | --- | --- | --- |
| **Clinical examination and evaluation** | | | |
| Toxicity assessments | X | X |  |
| The usage of drug support therapy  and expectant treatment | X | X |  |
| Physical examination | X | X | X |
| Digital rectal examination | X | X | X |
| Vital signs | X | X |  |
| ECOG performance status | X | X |  |
| **Sample collection for biomarker** | | | |
| Peripheral blood sample | Blood samples will be collected at 3 months, 1 year (if feasible), and 2 years (if feasible) after the day of the diagnosis of cCR and the beginning of watch and wait strategy | | |
| **Imaging evaluation** | | | |
| Enhanced CT, enhanced MRI and colorectal endoscopy | X | X | X |
| **Laboratory examination** |  |  |  |
| Blood routine examination | X | X |  |
| Biochemical test | X | X |  |
| Routine urine test | X | X |  |
| Thyroid function | X | X |  |
| Cardiac marker  (cardiac enzymes, troponin) | X | X |  |
| Survival follow-up and subsequent antitumor therapy |  | X | X |
| **Quality-of-life and rectal function assessments** | X | X (assessing at every 3 months in the first year and every 6 months in the second and third year) | |
